# Haemorrhagic versus non haemorrhagic ascites in cirrhosis: Their relationship and impact on prognosis of liver cirrhosis

**DOI:** 10.12669/pjms.36.4.2075

**Published:** 2020

**Authors:** Iftikhar Haider Naqvi, Khalid Mahmood, Abu Talib

**Affiliations:** 1 Dr. Iftikhar Haider Naqvi, FCPS. Department of Medicine, Dow University of Health Sciences, Karachi, Pakistan; 2 Prof. Khalid Mahmood, FCPS, FRCP(Edin), FRCP(Glasgow). Department of Medicine, Dow University of Health Sciences, Karachi, Pakistan; 3 Prof. Abu Talib, FCPS, FRCP(Glasgow). Department of Medicine, Dow University of Health Sciences, Karachi, Pakistan

**Keywords:** Ascites, Hemorrhagic, Cirrhosis, Portal hypertension, Spontaneous bacterial peritonitis

## Abstract

**Objectives:**

To evaluate the impact of haemorrhagic ascites on prognosis of patients with advance cirrhosis, this study was further aimed to assess the relationship between haemorrhagic ascites and advance cirrhosis and its effect on prognosis.

**Methods:**

Eight hundred and thirty-eight patients having liver cirrhosis with ascites were analyzed retrospectively (over three years) while segregated into two groups haemorrhagic and non haemorrhagic ascites. Patient outcome variables were identified among both groups and independent predictors for survival were analyzed. Kaplan-Meier survival estimates determined survival rate comparison between groups.

**Results:**

Haemorrhagic ascites was detected in (26.6%) patients. Spontaneous haemorrhagic ascites(79%) was the main cause of haemorrhagic ascites followed by hepatocellular carcinoma (14%) and iatrogenic (7.6%). Spontaneous bacterial peritonitis and acute kidney injury were statistically significant (p= 0.0001, 0.0001) among groups. Overall mortality at year three was higher (83%) in haemorrhagic ascites group. Survival among both groups (haemorrhagic versus non haemorrhagic) at one month, one year and three year was found to be significant (p= 0.000, 0.000 and 0.000).

**Conclusion:**

Haemorrhagic ascites impact overall survival with more mortality in comparison to non haemorrhagic ascites. Haemorrhagic ascites was an independent predictor of survival. Haemorrhagic ascites is possibly considered another predictor of survival among advance cirrhosis.

## INTRODUCTION

Cirrhosis is an end-stage liver disease having reported global prevalence of 4.5% to 9.5%.^1^,^2^ Cirrhosis of liver with its well-known complications, contributes significantly to overall mortality worldwide.

Ascites being most frequent complication of cirrhosis of liver is also the commonest reason for hospital admission in cirrhotic patients.^3^ Presence of ascites profoundly impacts survival of cirrhotic patients as evidenced by reported mortality of 15% and 44% within one and five year respectively.^4^ Hemorrhagic ascites defined as red blood cell (RBC) count greater than 10,000/mm³ against normal RBC count (< 1000/mm^3^) in ascitic fluid, is less frequent yet challenging complication among cirrhotic patients with ascites.^5^ Haemorrhagic ascites has 5% reported prevalence among cirrhotics with atypical features in comparison to usual ascites.^6^

Haemorrhagic ascites with its enhancing impact on morbidity and mortality of cirrhotic patients in relation to hepatocellular carcinoma, ruptured varices and trauma has been elaborated in earlier studies.^6^,^7^ The importance of routine ascitic fluid analysis in hospitalized patients with cirrhosis focuses on measuring white blood cell count to exclude spontaneous bacterial peritonitis. However, ascitic fluid analysis reveals RBC count < 50,000mm^3^ (between 10,000 to 50,000/mm3) among reasonable number of cirrhotic patients. The clinical utility of identifying haemorrhagic ascites, thus its impact on patients’ survival with advance liver disease is still undetermined on large scale. However, as consistently observed the patients with haemorrhagic ascites have a poor outcome and survival among patients with advanced cirrhosis of liver. Apart from two large retrospective studies,^8^,^9^ most reported data on haemorrhagic ascites were actually related to hemoperitoneum (RBCs before > 50,000/mm3) in non-critical clinical setting and described in small case series and case reports.^7^,^10^,^11^

Cirrhosis related Pakistani health statistics indicate huge increase in mortality from 10,324 (6,129–16,651) to 31,373 (16,325–61,028) within last three decades (from 1980 to 2010).^12^ The overall age-standardized mortality rate (per 100,000) in cirrhosis is 21.7% to 27.5% in Pakistan.^12^ Among well-known complications contributing to mortality in cirrhotics, data on haemorrhagic ascites is limited. This study was aimed to assess the relationship between haemorrhagic ascites and advance cirrhosis as well as its effect on overall impact on prognosis.

## METHODS

Retrospective data of 838 patients having confirmed cirrhosis of liver with ascites, were analyzed from January 2015 to December 2018 over three years. All patients aged ≥ 18 to 65 years of either sex having ascites who had at least one ascitic tap were enrolled at Medical Unit-1, Civil Hospital Karachi and Dow University of Health Sciences. Patients having malignancy, who had left against medical advice and with incomplete information, were excluded from study. Patients were segregated into two groups one haemorrhagic ascites where other was non haemorrhagic ascites group. Details of cirrhosis, its complications like hepatic encephalopathy (HE), hematemesis, portal vein thrombosis (PVT), hepatocellular carcinoma (HCC) and patients stay in high dependency unit were recorded. Investigations both base line and related to cirrhosis like haemogram, liver chemistries, International normalized ratio, creatinine, viral markers (HbsAg and Anti-HCV), ultra sound with splenic size and endoscopic data (Varices and their degree) were retrieved. Scores related to prognostication like Child Turcot Pugh score (CTP) and Model of End stage liver disease (MELD) score and death records of patients were also obtained from data.

### Cirrhosis

Cirrhosis of liver was confirmed on patient’s history related to cirrhosis, clinical features (ascites, hepatic encephalopathy and esophageal varices), imaging (ultrasonography and computed tomography showing small shrunken liver) and biochemical parameters. Histopathology also confirmed cirrhosis wherever required.^12^

### Hepatic encephalopathy

Hepatic encephalopathy and its various grades were labeled according to West Haven Criteria and graded 1-4.^13^

### Acute Kidney injury

Acute Kidney injury (AKI) is determined where ascites persists in cirrhosis even after withholding all diuretics and adequate fluid resuscitation whereas serum creatinine remained > 1.5 mg/dL.^14^

### Haemorrhagic ascites

Haemorrhagic ascites is defined when ascitic fluid contains >10,000/mm^3^ RBC as by earlier published data on the subject.^8^,^9^

### Non Haemorrhagic ascites

Non haemorrhagic ascites is defined when ascitic fluid contains < 10,000/mm^3^ RBC which is well in accordance to the earlier published data on the subject.^8^,^9^

### Causes of haemorrhagic ascites

1. Hepatocellular carcinoma (HCC) related

When advance imaging shows hemoperitoneum secondarily to HCC, including direct bleeding from mass, localized hematoma adjacent to mass, a liver mass ≥5 cm or mass of any size close to the surface (1 cm).^14^,^15^

### Iatrogenic hemorrhagic ascites

Hemoperitoneum detected in the patient after paracentesis, either diagnostic or therapeutic or liver biopsy.

### Spontaneous hemorrhagic ascites

Hemoperitoneum where no cause is identified.^16^,^17^

### Statistical Analyses

Data were analyzed through Statistical analyses SPSS software version 21 (SPSS Inc.; Chicago, Illinois, USA). Standard deviation and mean were used for descriptive analyses. Patients’ outcome variables were identified between haemorrhagic and non-haemorrhagic groups by univariate analysis and investigated through Chi square, Fisher exact, Student t and Mann-Whitney U tests, as required. Independent predictors for variables were analyzed by multivariate regression. Survival rate comparisons between both groups were determined using Kaplan-Meier survival estimates. To infer statistical significance A 5% type-I error level was used.

## RESULTS

### Demographic, clinical and biochemical profile

Out of 838 cirrhotic patients analyzed, haemorrhagic ascites was detected in 223(26.6%) patients whereas non haemorrhagic ascites was found in 615 (73.3%). Age, gender, aetiology of cirrhosis and its severity among groups are highlighted in Table-I. Liver chemistries like ALT, bilirubin, albumin and INR among both groups with their statistical significance (p values of 0.01,.0001, 0.0001 and 0.000) have shown in Table-I. Severe liver disease as evidenced by MELD and CTP score was found in the patients with haemorrhagic ascites where mean CTP score was 10±1.7 and 9.1±1 (p=0.000) and MELD score was 23.1±9 and 19.2±6 (p= 0.000) in both groups respectively as shown in Table-I.

**Table-I:** Comparison of demographic, clinical and biochemical parameters between hemorrhagic versus non hemorrhagic groups.

		Hemorrhagic Ascites (n=223)		Non-Hemorrhagic Ascites (n=615)		P value

		n	%	n	%	
Age		44.8±14.5		49±13.4		0.000
Gender	Female	86	39	232	38	0.443
	Male	137	61	383	62	
Etiology	AIH	11	5	32	5	0.1
	Alcoholic Hepatitis	4	2	11	2	
	Cryptogenic	2	1	7	1	
	Hemochromatosis	2	1	6	1	
	HBV	59	26	159	26	
	HCV	136	61	374	61	
	Wilson Disease	9	4	26	4	
Clinical features	Diffuse abdominal pain	65	29%	129	21 %	0.016
	Abdominal distension	118	53 %	141	23 %	0.000
	Unconsciousness	98	44%	153	25%	0.000
Stages of CTP	CTP-A	7	3	19	3	0.06
	CTP-B	78	35	271	44	
	CTP-C	138	62	325	53

*Biochemical parameter*		*Mean ± SD*		*Mean ± SD*		

	ALT iu/ml	68±6.9		55.5±6.2		0.01
	Creatinine mg/dl)	1.5±0.8		1.28±0.7		0.000
	Bilirubin mg/dL	6.1±0.3		4.8±0.3		0.000
	INR	1.8±0.4		1.5±0.3		0.000
	MELD Score	23.1±9		19.2±6		0.000
	CTP Score	10±1.7		9.1±1		0.000
	Hb% gm/dL	7.3±1.2		8.7±1.1		0.000
	WBC /mm3	8±1.3		6.4±3.4		0.000
	Platelets/mm3	121±29		127±49		0.062

### Portal hypertension indices and complications

Spleen had a mean size of 16±3cm in the haemorrhagic ascites group and 15±3 cm in controls with statistical significance (p=0.0001). Stage of ascites with their frequency among both groups have statistical significance (p=0.18) in Table-II. Degree of varices with their frequency among the groups having statistical significance (p=0.0001) Table-II. Various complications of cirrhosis among both groups showed only SBP and AKI to be statistically significant (p= 0.0001, 0.0001) as shown in Table-II.

**Table-II:** Comparison of Indices of portal hypertension and complications between hemorrhagic versus non hemorrhagic ascites groups.

		Hemorrhagic Ascites (n=223)		Non-Hemorrhagic Ascites (n=615)		P value

		n	%	n	%	
Splenic Size		16±3		15±3		0.0001
Stages of Ascites	Stage A	13	6	30	5	0.818
	Stage B	101	45	274	45	
	Stage C	109	49	311	50	
Degree of Varices	1^0^	47	21	123	20	0.0001
	2^0^	142	64	222	36	
	3^0^	34	15	270	44	
complications	Hepatic Encephalopathy Present	105	47	327	53	0.070
	Absent	118	53	288	47	
	Haemetemesis Present	174	78	534	87	0.002
	Absent	49	22	81	13	
	Portal vein thrombosis Present	185	83	519	84	0.344
	Absent	38	17	96	16	
	SBP Present	125	56	548	89	0.000
	Absent	98	44	67	11	
	AKI Present	103	46	418	68	0.000
	Absent	120	54	197	32	

### Causes of haemorrhagic ascites

Spontaneous haemorrhagic ascites 176 (79%) was the main cause of haemorrhagic ascites followed by HCC 30(14%) and iatrogenic 17(7.6%) in this study.

### Survival analysis

Overall mortality at year 3 was 83% in comparison to 70% among non haemorrhagic ascites. From the haemorrhagic ascitic group 71% survived one month, 17% survived 1 year and 13% patients survived 3 year with survival probability estimates (.73, 0.18 and 0.135) respectively. Whereas, from non haemorrhagic ascites group 87% survived one month, 50% survived 1 year and 27% patients survived 3 year with survival probability estimates (.87, 0.51 and 0.28) respectively was found significant (p= 0.000, 0.000 and 0.000) as shown in Fig-I.

**Fig.1:**
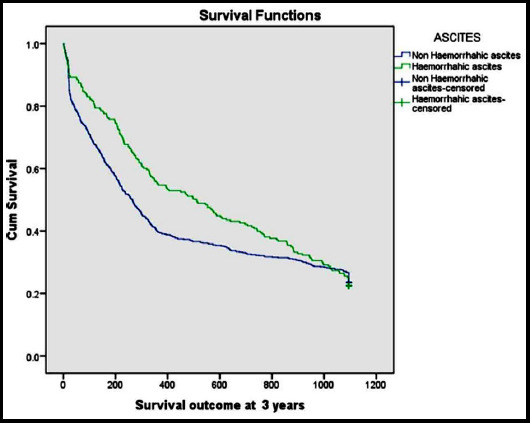
Survival outcome of haemorrhagic and non haemorrhagic ascites at 3 years.

### Predictors of mortality

Among various parameters only haemorrhagic ascites (Odd ratio=0.45,P=0.000, CI = 0.31-0.734), hepatic encephalopathy (Odd ratio=0.347,P=0.000, CI = 0.214-0.563) and SBP (Odd ratio 6.07, p=0.000,CI = 2.6-14.2) qualified as independent predictors of mortality. Table-III

**Table-III:** Determination of independent predictors of mortality (multinomial logistic regression analysis).

Variable	Odds Ratio	P Value	Confidence Interval
Age	1.00	0.216	0.997-1.021
Gender	1.143	0.449	0.809-1.614
Haemorrhagic ascites	0.45	0.000	0.31-0.734
MELD Score	0.994	0.645	0.969-1.019
Hepatic encephalopathy	0.347	0.000	0.214-0.563
Hematemesis	0.499	0.263	0.147-1.686
Portal vein Thrombosis	0.659	0.405	0.247-1.757
SBP	6.07	0.000	2.6-14.2
AKI	1.685	0.07	0.959-2.961

### Ascitic RBC’S range

Patients of haemorrhagic ascites were grouped on the basis of ascitic RBC’S count where 16 (7.3%) patients had ascitic RBC’S count > 50,000/mm3 while majority had ascitic RBC’S count between 10,000/mm3 – 50,000/mm3. Statistical significance is not evidenced as p values shown (0.73, 0.60, 0.32 and 0.80). Table-IV.

**Table-IV:** Comparison of complication of cirrhosis among subgroups of haemorrhagic ascites.

Complications of cirrhosis	Haemorrhagic ascites (RBC’S > 50,000/mm3 N (%)	Non haemorrhagic ascites (RBC’S 10,000-50,000/mm3) N (%)	P Value
HDU admission	10 (65%)	139 (67%)	0.73
AKI	07(43.7%)	109 (52.6%)	0.60
SBP	09(55%)	87(42%)	0.32
Hepatic encephalopathy	09 (55%)	108(52%)	0.80

## DISCUSSION

Haemorrhagic ascites was present in 223 (26.6%) in this study whereas earlier studies^8^,^9^ have 25% and 35.5% patients with haemorrhagic ascites. Most patients in this study had viral related (Chronic HCV and HBV) as the cause of cirrhosis whereas study by Yıldız et al.^9^ showed chronic HBV followed by HCV mainly causing cirrhosis. Urrangana et al.^8^ showed alcohol as a cause of cirrhosis followed by chronic HCV and HBV.

Hyponatremia, raised creatinine, hypotension and advance severity of liver disease (High CTP and MELD score) are well established poor prognostic indicators among patients with liver cirrhosis.^4^,^19^,^20^ Spontaneous hemorrhagic ascites was found incidentally among cirrhotics presents without signs of haemorrhage (hypotension, tachycardia and syncope). Earlier studies^6^,^10^ suggest that hemorrhagic ascites may indicate poor prognosis among cirrhotics due to increased risk of AKI, HE and high mortality. Two possible mechanisms related to development of spontaneous haemorrhagic ascites have been proposed.^10^ First proposed mechanism is of intra-abdominal bleeding from an organ or a small peritoneal vessel, or a varix,^13^ whereas second is related to raised portal or splenic pressure causing diapedesis of erythrocytes within peritoneum.

Increased splenic size and higher degree of varices in patients with haemorrhagic ascites in this study validates the role of raised portal or splenic pressure as a cause of haemorrhagic ascites. This is similar to the earlier studies.^6^,^10^ Complications like haemetemesis, AKI and SBP occur frequently with haemorrhagic ascites as compared to non haemorrhagic ascites. Earlier studies^8^,^9^ have also endorsed SBP and AKI as frequently reported problem with haemorrhagic ascites whereas HE was also found significantly.

This study showed high mortality rate at 1 month, 1 year and 3 year among patients with haemorrhagic ascites like large earlier published studies.^8^,^9^ This study has tested various determinants like Haemorrhagic ascites, HE, portal vein thrombosis, SBP as an independent predictor of mortality among patient of cirrhosis with ascites and found haemorrhagic ascites, SBP and HE as an independent predictor of mortality. Yildiz et al.^9^ had shown haemorrhagic ascites along with hepatorenal syndrome and HCC as an independent predictor for mortality in large cohort at Turkey. Urrunaga et al.^8^ in their study had also shown similar results where multilogistic regression determined haemorrhagic ascites as an independent predictor of mortality along with HCC and high MELD score.

Current study also tested range of ascitic RBC’S count among haemorrhagic ascites either having 10,000 – 50,000/mm3 or > 50,000/mm3 as earlier determined by Yildiz et al.^9^ and found same results. This further validates earlier study that 10,000/mm3-50,000/mm3 ascitic RBC’S count can be considered for haemorrhagic ascites. Among types of haemorrhagic ascites spontaneous haemorrhage was the most common cause in this study with abdominal distension. Haemorrhagic ascites presenting with worsening ascites and shock is always related to ruptured varices or HCC have been reported in about 0.5% patient.^10^,^6^,^21^ This study had shown 07 (3.1%) patients who died with HCC related haemorrhage which is quite high as compare to earlier study.^8^

### Limitations of the study

It was retrospective design and missing of iatrogenic hemorrhagic ascites at first paracentesis. However, imploring experienced physicians in paracentesis, making it ultrasound guided, including first paracentesis value and omission of two and 3^rd^ paracentesis values have overcome the problem. Even though ascitic tap related hemorrhage or bleeding complications of peritoneum is very rare (0.01%) like earlier studies.^23^,^24^

## CONCLUSION

Haemorrhagic ascites impact overall survival with more mortality in comparison to non haemorrhagic ascites. Haemorrhagic ascites was an independent predictor of survival. Haemorrhagic ascites is possibly considered another predictor of survival among advance cirrhosis.

### Authors Contribution

**IHN:** Conceived, designed and did statistical analysis & editing of manuscript.

**IHN** **& AT:** Did data collection and manuscript writing.

**KM:** Did review and final approval of manuscript.
